# Targeted detection of sequence variants in cell-free DNA from cerebrospinal fluid in pediatric central nervous system tumors

**DOI:** 10.3389/fonc.2024.1513073

**Published:** 2025-01-06

**Authors:** Katrina O’Halloran, Erin E. Crotty, Eirini Christodoulou, Sarah E. Leary, Alexandra Miller, Vera A. Paulson, Christina M. Lockwood, Ashley S. Margol, Jaclyn A. Biegel

**Affiliations:** ^1^ Cancer and Blood Disease Institute, Children’s Hospital Los Angeles, Los Angeles, CA, United States; ^2^ Keck School of Medicine, University of Southern California, Los Angeles, CA, United States; ^3^ Ben Towne Center for Childhood Cancer and Blood Disorders Research and the Department of Pediatrics, Seattle Children’s Hospital, Fred Hutchinson Cancer Research Center, University of Washington, Seattle, WA, United States; ^4^ Department of Pathology, Harvard Medical School and Brigham and Women’s Hospital, Boston, MA, United States; ^5^ Brain and Spine Tumor Center, Perlmutter Cancer Center, New York University Langone, New York, NY, United States; ^6^ Department of Neurology, New York University-Langone Health, New York, NY, United States; ^7^ Genetics and Solid Tumor Laboratory, Department of Laboratory Medicine and Pathology, University of Washington School of Medicine, Seattle, WA, United States; ^8^ Department of Pathology and Laboratory Medicine, Children’s Hospital Los Angeles, Los Angeles, CA, United States

**Keywords:** pediatric neuro-oncology, cerebrospinal fluid, liquid biopsy, targeted sequencing, sequence variant

## Abstract

The emergence of liquid biopsy technologies holds great promise in the cancer setting, including in pediatric central nervous system (CNS) tumors. In contrast to broad lower-depth sequencing, commonly referred to as low pass whole genome sequencing (WGS), targeted platforms with a higher depth of coverage have also been established. Here, we review targeted liquid biopsy techniques with applicability to pediatric CNS tumors. These include polymerase chain reaction (PCR), both droplet digital PCR and reverse transcription-based PCR, Sanger sequencing, and next-generation sequencing approaches that incorporate amplicon- and hybrid capture-based methods. The goal of this paper is to facilitate an understanding of these targeted techniques and provide a context for clinical relevance within disease categories, as well as a discussion on optimizing real-world implementation for pediatric CNS tumors.

## Introduction

### Pediatric central nervous system tumors

With the advent of molecular genomic technologies, disease-defining and oncogenic alterations have been identified across various cancers, including pediatric central nervous system (CNS) tumors. Increasingly, molecular findings that refine traditional histopathological diagnoses are being incorporated into diagnostic criteria (1–3). Medulloblastomas and other embryonal CNS tumors are largely defined by copy number alterations with larger scale gene or whole/partial chromosomal gains or deletions, whereas DNA sequence variants and fusion events are used to classify a variety of glial tumors. These findings can aid in prognostication by identifying clinically significant alterations such as *H3-3A* (formerly *H3F3A*) mutations, the hallmark alteration in a majority of diffuse midline gliomas (DMG), and *KIAA1549::BRAF* fusions commonly seen in pediatric low grade gliomas (LGG). Molecular characterization can also aid in treatment decisions when targetable alterations are identified. Agents that target the mitogen-activated protein kinase (MAP kinase) pathways, such as BRAF and MEK inhibitors, can be used in tumors harboring pathway alterations including a BRAFV600E point mutation or *KIAA149::BRAF* fusion, respectively.

### Liquid biopsy in pediatric CNS tumors

Successful detection of circulating tumor DNA (ctDNA) in CSF samples using genome-wide analysis has demonstrated the utility of CSF liquid biopsy for pediatric CNS tumors. Low-pass whole genome sequencing of CSF-derived cell-free DNA (cfDNA) was shown to be prognostic in patients with medulloblastoma (4–8). Nanopore sequencing is a long-read sequencing modality with initial applications in intraoperative molecular diagnostics, and more recent applications in CSF-derived cfDNA analysis (9, 10). In contrast to low-depth WGS or nanopore sequencing, targeted platforms with a higher depth of coverage have also been established for CSF-derived cfDNA-based liquid biopsy assays in pediatric patients with CNS tumors (5, 7, 11, 12). The potential for the clinical use of liquid biopsies is promising, with broad applications, particularly in patients with brain and spinal cord tumors where neurosurgical interventions can carry significant risks. Liquid biopsies may enable minimally invasive diagnoses, longitudinal monitoring over time, and early detection of relapse.

The goal of this review is to facilitate an understanding of these targeted techniques and provide a context for clinical relevance within pediatric CNS disease categories, as well as a discussion on optimizing real-world implementation for pediatric patients with CNS tumors.

## Methodologies

### Polymerase chain reaction-based approaches

#### Droplet digital PCR

Droplet digital PCR (ddPCR) is a rapid PCR method for the detection of known genetic alterations in a variety of cancer subtypes (13, 14). The highly sensitive and specific nature of ddPCR involves partitioning a sample into 20,000 individual droplets, prior to performing PCR and producing a fluorescent signal for the wild-type and mutant target of interest. The first clinically relevant target for ddPCR assays using liquid biopsies from pediatric patients with CNS tumors, was the detection of the BRAFV600E mutation (15). Arthur et al. showed that serum, plasma, and in some cases CSF-derived cfDNA could be successfully analyzed to detect the V600E mutation and guide therapy. A more recent report used ddPCR on CSF from a young child with an inoperable brainstem tumor to demonstrate a BRAFV600E mutation, enabling targeted treatment (16).

Additionally, the accurate mutant allele quantification of recurrent mutations in histone 3 (*H3-3A*, *H3C2, or H3C3*) at lysine position 28 (K28M), aka H3K27M, in patients with DMG has been shown, and the ability to detect driver mutations such as H3K27M with >75% detection rates in CSF highlights the potential diagnostic utility of these assays (17–21). Several studies have also confirmed longitudinal surveillance of DMG by analyzing multiple CSF specimens from patients over time to track the burden of the H3K27M mutation as a supplement to radiographic disease surveillance (17, 18, 22). A clinical trial from the Pediatric Neuro-Oncology Consortium (PNOC) demonstrated that plasma-derived ctDNA detection of H3K27M was successful in 92% of H3K27M mutant cases (23), further supporting the clinical utility of liquid biopsies to evaluate patients with DMG.

Evidence of measurable residual disease (MRD) and genomic evolution in medulloblastoma was reported by Escudero et al. who performed ddPCR of CSF from 13 patients (8). Variants identified by ddPCR included those in *BCOR*, *PTCH1*, *CTNNB1*, and *SRCAP*. A subsequent study by Kojic et al. described personalized somatic mutation ddPCR assays for multiple pediatric CNS tumor types including ependymoma, embryonal tumors, CNS neuroblastoma, and medulloblastoma (24). The detection of variants in multiple genes such as *PIK3IP1* and *CLIP2* using as little as 0.17ng of cfDNA, and 1ml of CSF demonstrate that sensitive and specific tumor burden surveillance can be performed with very small CSF volumes.

There are several limitations of ddPCR technology including the relative cost, which is increased when designing multiple assays, the limited number of targets that can be evaluated simultaneously, specific equipment, and time-consuming probe design that may impact assay specificity and widespread adoption. In certain cases, the primers designed for individual assays may match multiple regions in the genome limiting the analysis of multiple targets (25–27).

#### Reverse transcription PCR

Unlike ddPCR, reverse transcription PCR (RT-PCR) involves reverse transcribing a fragment of RNA to a complementary DNA strand, which is then amplified. RT-PCR has been successfully applied to evaluate serum miRNA profiles as a biomarker in pilocytic astrocytoma which was shown to be correlated with tumor volume (28, 29). In addition, the quantitative detection of miRNAs by RT-PCR was described for pediatric patients with malignant germ cell tumors, and results indicated a high diagnostic sensitivity and specificity, with the ability to distinguish germ cell versus non-germ cell intracranial tumors utilizing CSF (30).

Combined, ddPCR and RT-PCR have great potential in the clinical utility of liquid biopsies for pediatric patients with CNS tumors. Both RT-PCR – and particularly ddPCR - are sensitive quantitative approaches, while also being cost- and time-effective when processing large numbers of specimens at the same time. Furthermore, multiplexing PCR reactions allows for the detection of multiple targets of interest, and may be applied to both ddPCR and RT-PCR. Potential applications include aiding in diagnosis and in long-term monitoring by tracking the allele frequency of a disease-defining hotspot variant.

### Sanger and next-generation sequencing approaches

#### Sanger sequencing

Sanger sequencing is a highly accurate sequencing method that utilizes electrophoresis and chain-terminating dideoxynucleotides, sequencing one DNA fragment at a time (31). Huang et al. used both Sanger sequencing and nested PCR to evaluate CSF for *H3* mutations from patients with DMG (32). Sanger sequencing was used to detect an H3K27M mutation in the CSF of two of four patients with a diagnosis of DMG, and detected a G35V alteration in the CSF of a patient with a hemispheric glioma. The clinical application of Sanger sequencing is primarily for confirmation of suspected germline variants which may be detected with primary tumor or liquid biopsy-based sequencing. Sanger sequencing has the lowest sensitivity of all of the molecular methods, and requires larger amounts of DNA than that which is typically isolated from CSF.

#### Next generation sequencing

In contrast to Sanger sequencing, NGS methods involve parallel sequencing of millions of DNA fragments simultaneously, allowing for higher throughput (33). Most NGS assays employ short-read sequencing, which are amenable to ctDNA applications, and targeted panel-based designs which may be used to evaluate hundreds of genes of interest in a single assay. However, broad NGS panels may require increased amounts of input DNA, which can be a challenge given that the yield of CSF-derived ctDNA is generally low in these patients.

#### Hybrid capture-based methods

Hybridization-based capture methods for targeted NGS may utilize probes complementary to specific DNA targets and beads which isolate the probe-target complex, allowing for sequencing of a product enriched for DNA regions of interest. A primary advantage of the hybrid capture-based approach is that it can detect variants within the entire fragment, which is especially helpful in detecting gene fusions with variable partners. Various hybrid capture panels have been used to detect ctDNA in pediatric CNS tumors.

The Memorial Sloan Kettering-Integrated Mutation Profiling of Actionable Cancer Targets (MSK-IMPACT) panel has been most widely used (34). Initial studies of this panel using cfDNA, especially in the setting of CNS metastasis, demonstrated detection of oncogenic alterations in 47% of samples. A higher rate of ctDNA detection was associated with disseminated rather than focal disease (35). Prior work in adult patients with gliomas utilizing the MSK-IMPACT panel has also shown evidence for tumor evolution, with alterations noted primarily in growth factor receptor signaling pathways (11).

Pagès et al. recently published a prospective study with over 500 samples from more than 250 children with various CNS tumors, assessing not only CSF, but also plasma and urine, which was the largest study to date focused on pediatric patients (7). Alterations were detected in 3 of 10 CSF samples, but only high-grade tumors had detectable ctDNA in liquid biopsy samples. The LBSeq4Kids liquid biopsy-based assay at Children’s Hospital Los Angeles (CHLA) employs LP-WGS for detection of copy number alterations, and a custom hybrid gene capture panel that was initially focused on pediatric gliomas (5, 36). Mutations in cfDNA from the CSF were identified in 9 of 15 patients with primary brain tumors. Of note the *KIAA1549::BRAF* fusion was successfully identified in the CSF in 7 of 10 patients with primary tumors harboring these fusions. Similarly, the Seattle Children’s Hospital institutional experience utilizing the clinically validated UW-OncoPlex platform showed that cfDNA was successfully isolated in 9 of 17 CSF samples, including *PTCH1*, *SMARCB1*, and *H3F3A* (12). This was achieved with very small amounts of CSF; the median CSF volume was 0.4 mL with a range of 0.2 to 3 mL.

#### Amplicon-based NGS

Amplicon-based sequencing incorporates primers which are complementary to specific genomic sequences and utilizes PCR to generate products termed “amplicons” which may then be sequenced. Advantages include rapid turn around time and relatively lower cost when compared to hybrid capture-based methods. This modality has not been well established or widely used in ctDNA detection in CSF from children with CNS tumors.

In addition to the above technologies which have focused on cfDNA, additional potential applications include the analysis of DNA located in extracellular vesicles (EV), where DNA is relatively protected. EVs have been successfully evaluated in a number of pediatric CNS diaseases, including medulloblastoma and glioma (37, 38). Furthermore, the analysis of EVs and cfDNA are not mutually exclusive but potentially complementary, utilizing the aforementioned techniques. With the above, and ever-emerging new technologies and applications, an integrative approach is likely needed for comprehensive liquid biopsy assessment. A summary of relevant literature with regard to various cfDNA techniques may be found in **Table 1**.

**Table 1:** Literature on CSF liquid biopsy using various targeted sequencing techniques.

Study	Tumor types	Targeted sequencing method	Target(s)	Assay positivity (CSF)	Serial monitoring evaluated
PCR-based					
Stallard et al., 2018 (17)	Diffuse intrinsic pontine glioma (DIPG), glioblastoma multiforma (GBM)	ddPCR	H3-3A (H3F3A) mutations	4/4 patients (100%)	No (but multifocal at autopsy)
Panditharatna et al., 2018 (18)	Diffuse midline glioma	ddPCR	H3-3A (H3F3A) mutation (also *ACVR1*, *PIK3R1* or *BRAF multiplex*)	21/28(75%) at diagnosis	Yes
García-Romero et al., 2019 (15)	Various	dPCR	BRAFV600	CSF samples not available in 4 mutated primary tumors	No
Li et al., 2021 (19)	Diffuse midline glioma	ddPCR	H3-3A (H3F3A) mutation	Diagnosis in 6/6 (100%)	No
Cantor et al., 2022 (22)	Diffuse midline glioma	ddPCR	H3-3A (H3F3A) mutation	ctDNA detection in 28/29 CSF samples (97%)	Yes
Kojic et al., 2023 (24)	Various	ddPCR	Multiple recurrent driver mutations identified from WGS of primary tumor	Diagnosis in 11/12 (92%)	Yes
NGS-based					
Huang et al., 2017 (32)	Diffuse midline glioma	Sanger, nested PCR (mutation-specific primers)	H3-3A (H3F3A) H3C2 (HIST1H3B)	During treatment In 2/4 DIPG (50%) and 1 supra-tentorial GBM	No
Pan et al., 2015 (39)	Brainstem tumors	Panel (+ddPCR and amplicon)	Various	1/1 panel (100%) 6/7 (86%) ddPCR+amplicon	No
Wang et al., 2015 (46)	Various	Hybrid (+ WES)	Various	13/13 (100%) panel	No
Miller et al., 2019 (11) (adult)	Glioma	Hybrid	MSK-Impact	42/85 (48%)	Yes
Miller et al., 2022 (47)	Various	Hybrid	MSK-Impact	30/64 samples (47%)	Yes
Pagès et al., 2022 (7)	Various	Hybrid (and WGS)	OncoPanel	3/10 (30%) CSF	Yes
O’Halloran et al., 2023 (5)	Various	Hybrid (and WGS)	Custom panel	9/15 (60%)	No
Ronsley et al., 2024 (12)	Various	Hybrid	UW-OncoPlex	9/17 samples from 8/15 patients (53%)	Yes in 2 patients
Other					
Bruzek et al., 2020 (9)	High-grade glioma	Nanopore sequencing	Amplicon-based sequencing	Diagnosis in 108/127 (85%)	Yes

## Clinical utility

### Diagnosis

**Figure 1:**
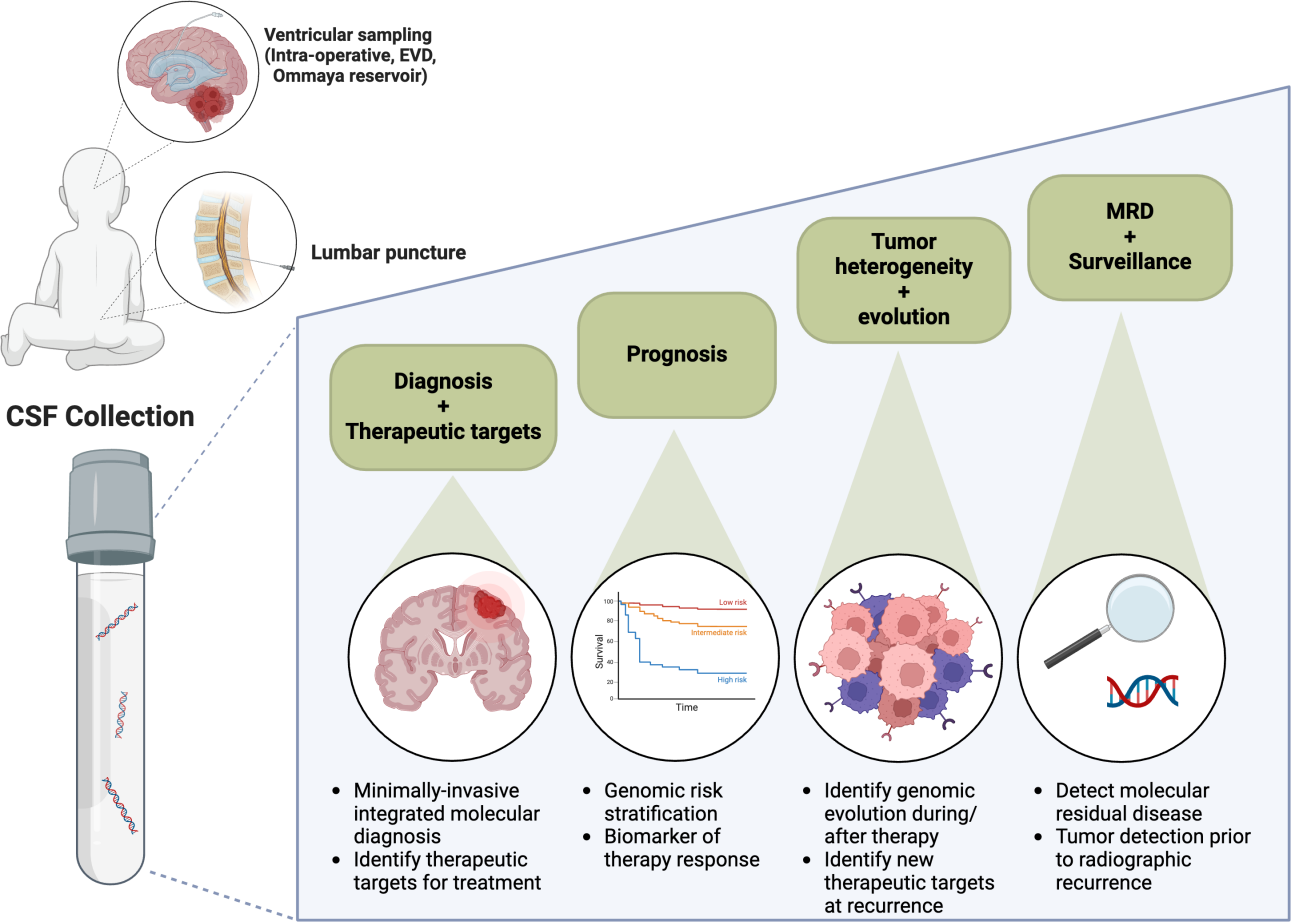
CSF liquid biopsy and future directions.

The potential clinical applications of liquid biopsy-based approaches are shown in **Figure 1** which highlights the current clinical applications and future directions for CSF liquid biopsy in pediatric CNS tumors. At the time of initial diagnosis, liquid biopsies may obviate the need for tissue and spare children the need for invasive surgery. In particular, tumors for which there is no role for surgical resection, for example diffuse brainstem tumors, may be uniquely suited to liquid biopsy-based approaches for tumor diagnosis (16, 17, 32). In addition, liquid biopsy specimens may better reflect tumor heterogeneity, as compared to often small primary tumor biopsies (39–41). Furthermore, liquid biopsies may be used to identify actionable molecular targets, including *PIK3CA* and *BRAF* variants (15, 16, 35). The latter, a BRAFV600 variant, has been successfully identified using liquid biopsy in a patient deemed inoperable and subsequently, the patient’s tumor demonstrated a response to BRAF targeted therapy (16).

### Monitoring

The utility of serial liquid biopsies has also been demonstrated in some clinical trials. One of the largest studies to date evaluated measurable residual disease in the setting of medulloblastoma by performing LP-WGS on CSF; those patients with a persistently positive liquid biopsy were shown to have a significantly higher risk of progression (4). While focused on LP-WGS, targeted sequencing of a limited panel of genes for baseline CSF samples was completed providing proof of concept for combined approaches in future studies.

Particularly in pediatric CNS tumors, where repeated invasive neurosurgical interventions may not be feasible, liquid biopsies can provide insight into tumor evolution. Liquid biopsies interrogated using the MSK-IMPACT panel closely resembled primary tumors molecularly with regard to mutations important in early tumorigenesis, for example *IDH* gene mutations, however, in serial sampling significant evolution was noted in growth factor signaling pathways (11).

Response to therapy can also be analyzed with liquid biopsy, as has been reported in patients with DMG. An early study evaluating liquid biopsy on a serial basis in patients undergoing treatment DMG showed that decreases in H3K27M ctDNA corresponded with radiographic tumor responses. ONC201 is an experimental impiridone, and several studies have now shown that ctDNA burden decreases in response to treatment with this novel agent (9, 22, 42). An early decrease in H3K27M was predictive of a longer progression-free survival time (22).

## Limitations

Current technologies have attempted to address the limitations, both technical and perceived, that may be impeding broad implementation of targeted CSF liquid biopsy platforms (43). For one, CSF as a biofluid is fairly acellular and sample volume is limited, particularly in children and adolescents. This impacts the cfDNA quantity for input into sequencing platforms, which can also be affected by additional preanalytic factors impacting both cfDNA quality and yield. In addition, CSF source may play a role, with potential differences between lumbar versus ventricular CSF, however, further evaluation is needed in order to better understand what these differences may be (35). Furthermore, technical factors such as type of collection tube, cfDNA stabilization after immediate centrifugation, storage temperature, and cfDNA isolation technique have also all been found to contribute to cfDNA yield (43, 44).

While clinical practices vary between centers, technology tends to be widely adopted after systematic analysis and optimization. However, this has yet to be achieved for pediatric CSF liquid biopsy-based assays. For example, both silica membrane columns and manual bead-based isolation kits are used for cfDNA recovery, with some groups showing one or the other kit to be superior (44, 45). However, many liquid biopsy protocols were originally designed and implemented for serum-derived cfDNA and require adaptation for the unique characteristics of CSF. CSF contains a paucity of cfDNA derived from non-tumor or alternative genomic sources, likely secondary to the effects of the blood-brain barrier and rarity of hematopoietic cells under normal physiologic conditions. This paucity of non-tumor cfDNA means that CSF is enriched for tumor DNA when compared to plasma and other biofluids (7, 40, 46). While relatively enriched, quantities of cfDNA in CSF remain low and the factors that influence rate of DNA shedding are largely unknown. Many groups have shown that CSF-derived ctDNA is more readily detected at diagnosis from patients with higher grade tumors, including high-grade glioma and medulloblastoma, compared to tumors with lower grade features (7). Contrast-enhancing disease and patients with leptomeningeal dissemination of disease are also found to have higher rates of detection (11). However, little is known about these mechanisms or potential vulnerabilities to exploit for optimization of cfDNA isolation. Properties inherent to the CSF compartment, the tumor itself, or preanalytical processing can also affect cfDNA input. As such, many platforms have focused on making improvements in downstream bioinformatic analyses in order to optimize results with minimal or suboptimal inputs. Historically, threshold quantities of cfDNA < 5 ng have been a barrier to confident variant calling, however newer cfDNA techniques are successful with lower cfDNA quantities for disease detection. Enzymatic methyl-seq (EM-seq; New England Biolabs) and other bisulfite-free methods, for example, can enable cfDNA methylation-based signatures from as little as 1 ng of isolated cfDNA.

Targeted sequencing methodologies typically require *a priori* knowledge of recurrent or driver genomic aberrations. Only a subset of pediatric CNS tumors will harbor canonical driver mutations (e.g. in Histone 3, *BRAF*, *NTRK*, *SMARCB1*) and the catalog of prognostic molecular findings is constantly evolving. A successful targeted CSF liquid biopsy approach will need to be nimble enough to adapt to new genomic discoveries over time. Furthermore, a combinatorial approach to testing has been shown by many groups to enhance ctDNA detection, for example in combination with LP-WGS, which may allow for excellent disease and mutation-agnostic surveillance (5, 7, 36).

Technology typically improves at a quicker pace than our ability to standardize laboratory methods and form a consensus on the clinical interpretation of a new test. The ideal scenario for incorporating a new screening or monitoring tool is to confirm optimal sensitivity, wherein the test identifies all individuals with tumor present without any false-negative results. In the case of an assay used to detect MRD, this is best done in the setting of a clinical trial. Efforts are currently ongoing to incorporate CSF liquid biopsy testing into upfront therapeutic trials in pediatric populations, where results can be evaluated in the context of centrally reviewed clinical information, including imaging and CSF cytology. Prior to the results of these trials, practitioners will need to be adept and cautious in assessing retrospective data on smaller cohorts of patients with careful attention paid to minimizing interpretation error.

## Future directions

With various proof-of-principle studies now published, as discussed in this manuscript, implementation of liquid biopsy-based technologies into clinical trials is underway and may serve as an opportunity to compare preanalytical factors such as CSF source (lumbar puncture, Ommaya reservoir, etc) and sequencing platform analytics. There are several ongoing studies with liquid biopsy aims that involve targeted sequencing for CSF-derived ctDNA detection. The Pediatric Brain Tumor Consortium (PBTC) N14 study seeks to determine the concordance of CSF and primary tumor profiling, using the MSK-IMPACT panel and importantly, liquid biopsy results will be performed in a CLIA-certified laboratory environment and thus will be shared with the treating medical team. NCT01106794 seeks to prospectively examine molecular profiles from CSF, serum, and urine in patients with diffuse intrinsic pontine glioma and other brainstem tumors. NCT05009992 (PNOC-022) is ongoing and assessing ONC201 in combination with additional targeted agents in a platform trial, and incorporates CSF liquid biopsy aims using nanopore sequencing. NCT04732065 (PNOC-023), which will evaluate ONC206 for patients with DMG, is collecting CSF for analysis using several different sequencing platforms. Several clinical trials using adoptive cellular transfer or other immunotherapy (e.g. CAR T cells, NK cells, PEP-CMV antigen vaccines, rHSC-DIPGVax vaccine, etc) also plan to collect CSF liquid biopsies for the evaluation of immune biomarkers and disease monitoring (NCT04185038, NCT05768880, NCT04196413, NCT04943848, NCT05096481, NCT05887882). Each of these trials will illuminate the utility of CSF-derived cfDNA in a particular population of pediatric or young adult patients with CNS tumors and in the context of a particular therapy. Taken together, these data will provide information on the broad applicability of this genomic technology and the potential for future clinical applications.

Beyond new therapeutics, there may also be a role for targeted CSF liquid biopsy-based assays to determine prognostic risk groups in patients receiving standard of care therapies. In pediatric medulloblastoma, CSF-derived MRD detected at the end of therapy by LP-WGS correlated with inferior PFS and OS, as previously described (4). Building off of this retrospective experience, prospective validation of CSF liquid biopsy in infants with medulloblastoma is being implemented into upfront clinical trials, and patients with positive MRD may be candidates for additional chemotherapy or radiation. Within this context, we can investigate whether CSF liquid biopsy-based assays can be employed for MRD monitoring, with the goal of escalating therapy for those at high risk of recurrence and minimizing therapy in patients with promising outcomes.

For routine clinical use, one of the biggest challenges in propelling CSF liquid biopsy forward is the lack of consensus on whether and when to obtain CSF for disease monitoring. In the framework of a clinical trial, CSF may be more readily obtained, so at a minimum CSF liquid biopsy should be incorporated into the correlative investigations of all new targeted and immunomodulating therapies. However, outside of trial assessments, there is no standard interval for performing a lumbar puncture procedure to obtain CSF during or after therapy. The Response Assessment in Neuro-Oncology (RANO) group has proposed recommendations for CSF liquid biopsy in adult glioma, but there is opportunity for adapting these recommendations towards special pediatric considerations, such as sample and biopsy limitations (47). Technically, targeted sequencing platforms that optimize flexible read lengths on short fragment cfDNA and that are validated for low DNA input will be more broadly adopted. It will also be important to determine which technologies may be optimal in various clinical contexts. For example, panel-based designs may be best in the setting of initial diagnosis of variant-driven tumors or monitoring for evolution as demonstrated by the acquisition of new sequence variants. In contrast, quantitative PCR-based approaches interrogating specific hotspots of interest may be best in evaluating response to therapy.

## Conclusions

The goal of this review has been to provide a comprehensive yet approachable evaluation of targeted sequencing technologies that are currently available. Applications specific to pediatric CNS tumors have been presented with a view and emphasis on their clinical relevance. As described, several pilot and proof-of-principle studies have now been completed with excellent results. The challenge for the medical and academic communities will be to determine how and when to best implement liquid biopsy-based methods into clinical care to optimize patient outcomes.
